# Hydroxyapatite-Complexed Type I Collagen and Fibrinogen-Modified Porous Titanium Alloy Scaffold: Promoting Osteogenesis and Soft Tissue Integration

**DOI:** 10.3390/mi16060692

**Published:** 2025-06-09

**Authors:** Wenhao Tao, Gang Tian, Xu Han, Jianyong Gao, Yingchun Zhu, Xiaogang Xu

**Affiliations:** 1 Department of Stomatology, First Affiliated Hospital of Naval Medical University, Shanghai 200433, China; twhsmmu@126.com (W.T.); tgsmmu@126.com (G.T.); hanxu8008@sina.com (X.H.); yongjiangao1976@126.com (J.G.); 2 Key Laboratory of Inorganic Coating Materials of Chinese Academy of Sciences, Shanghai Institute of Ceramics, Chinese Academy of Sciences, Shanghai 200050, China

**Keywords:** 3D printing, porous titanium alloy, polydopamine, osteogenesis, soft tissue integration

## Abstract

Titanium and its alloy scaffolds are widely utilized in clinical settings; however, their biologically inert surfaces and inherent mechanical characteristics impede osteogenesis and soft tissue integration, thereby limiting their application. Selective laser melting (SLM) was employed to fabricate scaffolds with matched cortical bone mechanical properties, achieving a composite coating of hydroxyapatite complexed with trace elements of silicon, strontium, and fluoride (mHA), along with type I collagen (Col I) and fibrinogen (Fg), thus activating the scaffold surface. Initially, we utilized the excellent adhesive properties of dopamine to co-deposit mHA and polydopamine (PDA) onto porous Ti-6Al-4V scaffolds, which was followed by immobilization of type I collagen and fibrinogen onto PDA. This bioinorganic/bioprotein composite coating, formed via PDA bonding, exhibits excellent stability. Moreover, in vitro cell experiments demonstrate excellent biocompatibility of the porous Ti-6Al-4V scaffold with composite bioactive coatings on its surface. Preosteoblasts (MC3T3-E1) and human keratinocytes (HaCaT) exhibit enhanced adhesion and proliferation activity, and the osteogenic performance of the scaffold is significantly improved. The PDA-mHA-Col I-Fg composite-coated porous titanium alloy scaffold holds significant promise in enhancing the efficacy of percutaneous bone transplantation and requires further investigation.

## 1. Introduction

The mandible, the only bone in the face with mobility, plays a crucial role in maintaining facial contour and structure. It significantly contributes to human mastication, food intake, and speech articulation. The loss of mandibular bone can impair both facial aesthetics and normal oral function to varying degrees [1]. Although traditional autologous bone transplantation stands as the gold standard for mandibular bone transplantation, the limited availability of autologous bone sources and donor complications restrict its application [2]. Hence, the quest for alternative scaffold materials to replace autologous bone remains crucial. Presently, Ti-6Al-4V has emerged as the primary material for scaffolds and other medical devices due to its exceptional biocompatibility, corrosion resistance, and mechanical properties [3]. Additionally, its well-developed processing technologies enables the feasibility of customized individual mandibular bone scaffolds [4]. However, conventionally manufactured titanium alloys, with significantly higher stiffness and modulus than normal bone, lead to issues such as stress shielding, aseptic implant loosening [5], bone nonunion, bone resorption, plate exposure [6], resulting in the failure of mandibular bone defect repair. To address these challenges, researchers have initially employed 3D printing to manufacture Ti-6Al-4V porous alloy scaffolds, ensuring biological safety [7] and ameliorating the high stiffness and elastic modulus issues associated with traditional titanium alloys, thereby rendering their mechanical properties more akin to those of natural bone tissue. Furthermore, the foam-like or mesh-like structures of such scaffolds promote osteoblast adhesion, proliferation, mineralization, and protein synthesis [8], positioning 3D printed Ti-6Al-4V porous alloy scaffolds as a potential alternative to traditional titanium and its alloy scaffolds. Nevertheless, the surface of pure titanium alloy scaffolds still exhibits biological inertness. Therefore, enhancing the biological performance of titanium alloy scaffolds necessitates surface modification.

A diverse array of strategies is currently under investigation to modulate the bioactivity of metallic surfaces, for instance, by tailoring their surface topography and chemistry [9,10,11]. Nevertheless, such monofunctional approaches often fall short of addressing the intricate challenges associated with suboptimal soft tissue integration. Bifunctional or multifunctional coatings, fabricated through specific surface modification techniques, are designed to concurrently impart two or more predefined functionalities to the implant surface. The fundamental principle of this strategy is to transcend the limitations of single-function designs, thereby enabling implants to effectively counteract the multifaceted challenges encountered within complex biological environments. Consequently, metallic implants at the soft tissue interface would significantly benefit from engineered surfaces possessing multifunctional properties that are capable of promoting organized soft tissue integration while concurrently preventing bacterial colonization, mitigating tissue inflammation, and averting implant loosening and eventual failure [12].

More than a decade had passed since 2007, when polydopamine (PDA) became a novel material with global significance and attracted widespread scientific research attention [13]. Research on polydopamine continues to rapidly expand, with broad applications in various fields such as chemistry, biomedicine, energy, environment, and materials science. This is attributed to its versatility in chemically modifying virtually any inorganic or organic material surface, enabling the construction of multifunctional coatings and providing secondary reactivity by facile chemical conjugation with target molecules [14]. Moreover, it is characterized by simple preparation processes, good biocompatibility, and strong adhesion [15]. Studies have found that PDA-modified nano-hydroxylapatite/chitosan composite hydrogel microspheres with strontium enhanced their biological performance [16]. However, PDA itself does not significantly enhance the biological performance of titanium alloy scaffolds; it mainly acts as an adhesive.

Hydroxyapatite (HA) is the main inorganic component of human bone, which provides strength, rigidity, and biocompatibility to bone tissue. Due to its biocompatibility, bioactivity, and ability to support bone cell attachment and growth, HA is an excellent choice for dental implants [17]. Gao et al. synthesized multi-element doped hydroxyapatite (mHA) containing trace amounts of silicon, strontium, and fluoride using a simple hydrothermal method. Their in vitro experiments demonstrated that mHA significantly promotes differentiation and osteogenesis of preosteoblasts (MC3T3-E1) and exhibits certain antimicrobial properties. This suggests that multi-element doping modification renders artificially synthesized hydroxyapatite closer to the inorganic components of human bone and possesses excellent osteoconductive properties, making it a promising coating material for oral implants [18].

Multi-doped HA addresses the issue of scaffold osteogenic capability, while collagen and fibrinogen are ideal choices for addressing poor bonding between the scaffold and soft tissues. Collagen is almost distributed in all tissues. More than 90% of the organic component in bone is type I collagen [19]. It provides a mechanically stable network for the extracellular matrix and regulates cellular responses through biological signaling. Furthermore, collagen-based biomaterials rarely elicit rejection reactions and lack fibrous encapsulation [20]. Inspired by mussel-inspired chemistry, Zhu et al. successfully covalently bound Type I collagen to titanium alloy coatings containing polydopamine (PDA), enhancing the stability of collagen on metal surfaces. Experimental evidence suggests that the addition of collagen promotes fibroblast adhesion, resulting in increased fibrinogen polymerization and improved cell spreading, all of which are crucial signals for wound healing at the implant-soft tissue interface [21].

The concept of enhancing bone formation and osteointegration by immobilizing biomolecules integral to osteogenesis onto metallic surfaces is scientifically sound. For instance, biomolecules such as type I collagen and fibrinogen have been successfully immobilized on titanium (Ti) surfaces [22].

Fibrinogen, through high binding affinity for various growth factors and integrins, provides important adhesion sites for fibroblasts and endothelial cells [23]. Thus, fibrinogen is suitable for improving soft tissue integration. Wang et al. prepared coatings containing fibrinogen on Ti-6Al-4V surfaces using PDA, demonstrating significantly enhanced bone growth and markedly reduced soft tissue reactions, as well as promoted angiogenesis [24]. Studies indicate that fibrinogen is critical for soft tissue repair and is one of the important substances for improving the bonding between implants and soft tissues.

**Figure 1:**
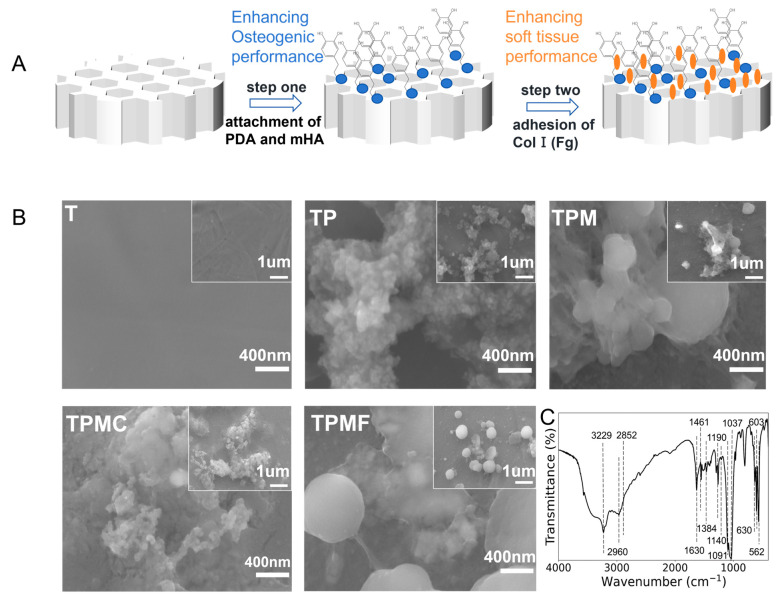
(**A**) schematic diagram of the coating preparation process. (**B**) Surface scanning electron microscope images of the five groups of samples:3D Printed Ti-6Al-4V Scaffold (T); T-PDA (TP); T-PDA-mHA (TPM); T-PDA-mHA-Col I (TPMC); T-PDA-mHA-Fg (TPMF). (**C**) FTIR waveforms of the TPM group.

In this study, a customized Ti-6Al-4V mesh scaffold was fabricated using 3D printing, and the scaffold surface was activated with silicon, strontium, and fluoride-doped hydroxyapatite, Type I collagen, and fibrinogen, as schematically illustrated in Figure 1A. To the best of our knowledge, based on the literature review, there are few reports on the combined modification of porous titanium alloy scaffolds with mHA and Type I collagen or mHA and fibrinogen. Existing scaffold surface modifications have predominantly focused on the osteogenic properties of the scaffold, neglecting the issue of soft tissue integration.

It is hypothesized in this study that the multi-element doped hydroxyapatite, which modified the surface of the widely used Ti-6Al-4V scaffold, along with Type I collagen and fibrinogen, will enhance the osteogenic and soft tissue sealing properties of the implant. Human keratinocyte (HaCaT) and preosteoblasts (MC3T3-E1) cells, relevant to soft tissue integration or osteogenesis, were cultured to evaluate cellular responses to the modified surfaces.

## 2. Materials and Methods

### 2.1. Fabrication of 3D Printed Ti-6Al-4V Scaffold and Composite Coatings

#### 2.1.1. Preparation of 3D Printed Ti-6Al-4V Scaffold

Following Li’s study [8], the Ti-6Al-4V was lightweighted using a rhombic dodecahedron structure, and porous Ti-6Al-4V scaffolds were manufactured using selective laser melting.

The Gibson-Ashby equation describes the preparation of the scaffold mesh structure:

$$\left[\frac{E}{E_{s}}={\left(\frac{\rho }{\rho_{s}}\right)}^{2}\right]$$

where *E* is the elastic modulus of the manufactured structure with density *ρ*, and $E_{s}$ and $\rho_{s}$ correspond to the elastic modulus and density of the respective solid. The percentage of porosity is also related to the relative density (*ρ/*$\rho_{s}$):

$$Porosity\left(\%\right)=(\left[1-\left(\rho /\rho_{s}\right)\right]\times 100)$$

For the Ti-6Al-4V (Chengdu Youcai Technology Co., Ltd. Chengdu, China) used herein, $E_{s}$ and $\rho_{s}$ are 110 GPa and 4.42 g/cm^3^, respectively.

Following the aforementioned procedures, the obtained samples are denoted as T. Subsequently, T undergoes cleaning and disinfection procedures, immersed in detergent for 1 h with ultrasonic circulation, where the detergent sequence includes acetone, isopropanol, ethanol, and deionized water. The scaffold is then subjected to high-pressure steam sterilization and dried overnight under ultraviolet light for future use.

#### 2.1.2. Preparation of Multi-Element Doped Hydroxyapatite

Following the prior research outcomes of the research group [18], Ca(OH)_2_ and Ca(H_2_PO_4_)_2_·H_2_0 were utilized as the primary reactants, while SiO_2_, SrO_2_, and CaF_2_ served as the sources of trace elements silicon, strontium, and fluorine for doping. Through hydrothermal synthesis, multi-element doped hydroxyapatite (mHA) was prepared to match the silicon, strontium, and fluorine content found in natural bone hydroxyapatite.

#### 2.1.3. Adhesion of Polydopamine and Multi-Element Doped Hydroxyapatite

The processed T samples were placed into a 48-well plate and reacted with the specified reagents according to Table 1.

The reaction proceeded in a constant temperature shaking incubator (NSKY, Shanghai, China) at 37 °C and 150 rpm for 24 h. After completion, the samples were washed three times with deionized water and dried for subsequent use. The resulting samples were respectively designated as T-PDA (TP) and T-PDA-mHA (TPM).

#### 2.1.4. Adhesion of Fibrinogen Coating

To couple fibrinogen (Fg) onto TPM, TPM was incubated in a 0.1 mg/mL fibrinogen (Sigma, San Diego, CA, USA) solution along with phosphate-buffered saline (PBS, hyclone, Logan, UT, USA) at 4 °C for 24 h, yielding T-PDA-mHA-Fg, denoted as TPMF. Subsequently, the samples were rinsed three times with PBS and ultrapure water and then dried with nitrogen gas. All samples were stored at 4 °C.

#### 2.1.5. Adhesion of Type I Collagen Coating

To couple Type I collagen onto TPM, TPM was incubated overnight in a 1.0 mg/mL type I collagen (Sigma, San Diego, CA, USA) solution at 4 °C, allowing collagen molecules to bind to the surface. After incubation, the samples were washed three times with deionized water to remove physically absorbed collagen I and dried the Scaffolds with the stream of nitrogen gas, resulting in T-PDA-mHA-Collagen I, denoted as TPMC. All samples were stored at 4 °C.

### 2.2. Testing and Characterization

#### 2.2.1. Surface Morphology Observation of Samples

The surface morphology of samples T, TP, TPM, TPMF, and TPMC was observed using a field emission scanning electron microscope (Ultra-55, Zeiss, Oberkochen, Germany) at magnifications of ×5000 and ×50,000.

#### 2.2.2. Static Water Contact Angle Measurement on Sample Surfaces

Due to the difficulties in hydrophilicity testing of porous scaffolds, smooth titanium alloy scaffolds fabricated by 3D printing were prepared according to the aforementioned method and tested after coating. Four groups were designated as Ts, Ts-PDA-mHA, Ts-PDA-mHA-Fg, and Ts-PDA-mHA-Col I. The static water contact angles on the surfaces of samples Ts, Ts-PDA-mHA, Ts-PDA-mHA-Fg, and Ts-PDA-mHA-Col I were measured using an SL200C optical dynamic/static water contact angle meter (Boston, MA, USA). In this process, the θ/2 method was employed, with 2 μL of water dropped onto each sample surface. Each sample was measured at four different points, with three repetitions at each point, and recorded in fully automatic trigger mode. The measurement results were reported as mean ± standard deviation. Statistical analysis was performed using a one-way analysis of variance (ANOVA) and least significant difference (LSD) test. Significance was considered at *p* < 0.05.

#### 2.2.3. Analysis of Surface Chemical Composition of Samples

The surface coatings of TPM were analyzed using Fourier-transform infrared spectroscopy (FTIR, IRAffinity-1, Kyoto, Japan). The scanning wavenumber ranged from 400 cm^−1^ to 1400 cm^−1^ with a scanning speed of 4 cm^−1^/s. The preparation of test samples involved scraping an appropriate amount of coating powder from the TPM surface. The PDA coating powder was then thoroughly mixed with dried potassium bromide powder at a mass ratio of 1:100 and pressed into thin sheets. X-ray photoelectron spectroscopy (XPS, Thermo ESCALAB 250Xi, Waltham, MA, USA) was employed to analyze the surface chemical elements of TPM, TPMF, and TPMC. Parameters were set as follows: scan calibration range from 0 to 1200 eV, power of 150 W, and binding energy calibrated to C1s at 284.8 eV. Avantage software v5.9921 was used for spectral deconvolution to calculate the elemental composition. Additionally, X-ray diffraction analysis (XRD, Ultima IV, Akishima City, Tokyo, Japan) was conducted to examine the phase composition of mHA powder and the surfaces of samples T, T-PDA, TPM, TPMF, and TPMC. The detection parameters were set as follows: operating voltage of 40 kV, operating current of 40 mA, scanning speed of 5°/min, and scanning angles ranging from 10° to 80°.

### 2.3. Coating Stability Assessment

To evaluate the stability of the PDA-mHA-Col I and PDA-mHA-Fg coatings, TPMC, TPMF, Ts-PDA-mHA-Col I, and Ts-PDA-mHA-Fg samples were immersed in PBS and incubated at room temperature for 7 days. Subsequently, the incubated samples were rinsed with deionized water and dried with a nitrogen stream labeled as TPMC-7d, TPMF-7d, Ts-PDA-mHA-Col I-7d, and Ts-PDA-mHA-Fg-7d. The difference in static water contact angles before and after immersion was characterized for Ts-PDA-mHA-Col I-7d and Ts-PDA-mHA-Fg-7d samples, and XPS elemental analysis was conducted on TPMC-7d and TPMF-7d samples.

### 2.4. In Vitro Cell Experiments

#### 2.4.1. Preparation of Experimental Samples

To ensure the sterility of samples T and TPM, sterilization was carried out using high-pressure steam. Subsequently, sterilized TPM was soaked in separately filtered and sterilized type I collagen and fibrinogen solutions to produce TPMC and TPMF, ensuring their freshly prepared status. In the experimental design, samples were divided into four groups, with 3D printed porous titanium alloy scaffolds (T group) serving as the control group, while TPM, TPMC, and TPMF samples constituted the three experimental groups.

#### 2.4.2. Cell Culturing

HaCaT cells were cultured in Dulbecco’s Modified Eagle’s Medium (DMEM, Gibco, Grand Island, NY, USA) supplemented with 10% fetal bovine serum (FBS, Gibco, Grand Island, NY, USA), 100 U/mL penicillin (Gibco, Grand Island, NY, USA), and 100 μg/mL streptomycin (Gibco, Grand Island, NY, USA). MC3T3-E1 were cultured in α-minimum essential medium (α-MEM, Gibco, Grand Island, NY, USA) supplemented with 10% FBS, 100 U/mL penicillin, and 100 μg/mL streptomycin. The cells were maintained at 37 °C in a humidified atmosphere with 5% CO_2_ and passaged when cell confluence reached 80–90%. HaCaT cell culture media were refreshed daily, while MC3T3-E1 cell culture media were refreshed every 2–3 days.

#### 2.4.3. Tissue Compatibility Testing

Cytotoxicity Experiment: Sample extraction preparation: According to the national standard GB/T 16886.12-2017 [25], two sets of samples were placed in a 24-well plate, with 2 pieces in each well, and 1 mL of complete culture medium was added as the extraction medium. The plate was then immersed in a 37 °C constant temperature culture oscillator for 24 h to collect the extraction solution, which was stored in a refrigerator at 4 °C for later use.

Well-cultivated HaCaT cells were seeded in a 96-well plate at a concentration of 8 × 10^4^ cells/mL, with 150 μL of cell suspension added to each well. The plate was then placed in a constant temperature incubator for 24 h until the cells completely adhered to the wall. The original culture medium was aspirated, and the cells were gently washed three times with PBS solution. Then, 150 μL of extraction solution at different concentration gradients (diluted with complete culture medium to 10%, 50%, and 100% concentrations, respectively) was added to each well, with a blank control group of 100% complete culture medium set for each plate. After 24 h of incubation in a constant temperature incubator, the original culture medium was aspirated, and 150 μL of PBS solution was added to each well. The growth of cells in each group was observed and photographed under an inverted fluorescence microscope (IX71, OLYMPUS, Tokyo, Japan), and cell viability was determined using the CCK-8 assay, with absorbance values measured at a wavelength of 450 nm using an enzyme-linked immunosorbent assay reader.

#### 2.4.4. Cell Adhesion and Spreading Experiment

The sterilized four groups of samples were placed in a 24-well plate. MC3T3-E1 cells were seeded onto the substrates at a density of 3 × 10^4^ cells/well and cultured for 12 h and 24 h. HaCaT cells were seeded at a density of 4 × 10^4^ cells/well and incubated for 12 h and 24 h. Cells were stained for F-actin and nuclei to observe cell adhesion. After specific incubation times, cells were gently washed with PBS, fixed with 4% paraformaldehyde for 15 min at room temperature, and permeabilized with 0.1% Triton X-100 for 5 min to enhance membrane permeability. Between each step with new reagents, samples were rinsed with PBS to optimize the subsequent reagent’s efficacy. For F-actin visualization, all samples were stained with 1 μg/mL fluorescein isothiocyanate-labeled phalloidin (FTIC-Phalloidin) for 30 min at room temperature. Subsequently, nuclei were stained with 5 μg/mL 4′,6-diamidino-2-phenylindole (DAPI). Finally, all samples were thoroughly washed with PBS three times to remove excess fluorescent reagents. Fluorescently stained cells were observed and photographed using an inverted fluorescence microscope (IX71, OLYMPUS, Tokyo, Japan). Cell adhesion was assessed at 12 h and 24 h using the CCK-8 assay, with absorbance measured at 450 nm using an enzyme-linked immunosorbent assay reader (Multiskan FC, Shanghai, China).

#### 2.4.5. Cell Proliferation Experiment

The sterilized four groups of samples were placed in a 24-well plate. MC3T3-E1 cells were seeded onto the substrates at a density of 3 × 10^4^ cells/well and cultured for 1, 4, and 7 days. HaCaT cells were seeded at a density of 4 × 10^4^ cells/well and incubated for 1, 4, and 7 days. Subsequently, cell proliferation status was assessed at 1, 4, and 7 days using the CCK-8 assay, with absorbance values measured at a wavelength of 450 nm using an enzyme-linked immunosorbent assay reader.

#### 2.4.6. Alkaline Phosphatase Activity Assay

Three groups of MC3T3-E1 cells (3 × 10^4^ cells) were seeded onto T, TPM, TPMC, and TPMF samples, and alkaline phosphatase (ALP) activity was measured at 4, 7, and 10 days, respectively. Cells were lysed in 300 μL of Tris HCl (10 mM) containing 0.1% Triton X-100 at −20 °C for 10 min. Subsequently, 50 μL of the supernatant from each sample was mixed with 100 μL of ALP assay reagent and incubated at 37 °C for 30 min. The absorbance of the mixture was then measured at 405 nm using a spectrophotometer. Total protein content was determined using a BCA assay kit. Relative ALP activity was obtained by standardizing the absorbance values with total protein content (OD/mg total protein).

#### 2.4.7. Measurement of Osteogenic Gene Expression Levels

Osteogenic gene expression levels of alkaline phosphatase (ALP), osteocalcin (OCN), and Runx-related transcription factor 2 (Runx2) were determined using real-time quantitative PCR (qPCR). MC3T3-E1 cells (3 × 10^4^ cells/well) were seeded on T, TPM, TPMC, and TPMF samples and cultured for 14 days. Total RNA was extracted using Trizol reagent (Sigma), followed by cDNA synthesis using a PCR kit (Takara, Beijing, China). Gene expression was assessed through qRT-PCR using the StepOne™ real-time PCR system (Applied Biosystems, Waltham, MA, USA). GAPDH was used as the housekeeping gene, and the gene primers utilized are detailed in Table 2.

#### 2.4.8. Alizarin Red Staining (Late-Stage Detection of Cell Osteogenic Differentiation)

MC3T3-E1 cells (3 × 10^4^ cells) were seeded on T, TPM, TPMC, and TPMF samples and cultured in a 37 °C, 5% CO_2_ humidified incubator for 14 days. After the 14-day culture period, the cell culture medium was removed, and the cells were washed three times with phosphate-buffered saline (PBS). Subsequently, 1 mL of 4% paraformaldehyde fixative was added to each well and incubated at room temperature for 1 h. The paraformaldehyde solution was then removed, and the cells were washed three times with PBS. Alizarin Red S staining solution was added, and the cells were stained in the dark at room temperature for 30 min. After removing the staining solution, the cells were washed five times with PBS until the washout became clear and the liquid in the wells was thoroughly aspirated. Finally, the samples were examined, and the staining of the cells was documented under an inverted microscope.

### 2.5. Statistical Analysis

All experiments were performed in six replicates, and the data are presented as mean ± standard deviation. Statistical analysis was conducted using ANOVA. *p* < 0.05 was considered statistically significant.

## 3. Results

### 3.1. Preparation of 3D Printed Porous Titanium Alloy Scaffolds and Composite Coatings

Porous Ti-6Al-4V mesh scaffolds with designed size were fabricated using selective laser melting technology. According to the Gibson-Ashby equation, the porosity of the scaffolds is 56.0% ± 2.0%, and the elastic modulus ranged from 19.4 to 23.3 GPa, approaching the elastic modulus of hard cortical bone. Following the process illustrated in Figure 1A, the 3D-printed Ti-6Al-4V was modified using mHA and Col I (Fg).

### 3.2. Surface Characterization of the Samples

#### 3.2.1. Surface Morphology Observation

Surface morphology was examined using field emission scanning electron microscopy (FE-SEM) (Figure 1B). The T group exhibited a relatively smooth surface, while clustered polydopamine (PDA) aggregates were visible on the surface of TP. Spherical mHA particles and PDA agglomerates were observed on the surface of TPM, with scattered mHA also present. Following the Col I modification, the surface of sample TPMC displayed a covering of type I collagen. Upon Fg modification, sample TPMF exhibited filamentous structures between spherical mHA, with PDA agglomerates covered by a thin film.

#### 3.2.2. Surface Chemical Composition Analysis

Fourier transform infrared spectroscopy (FTIR) was employed for elemental analysis of the surface coatings of the TPM samples. As depicted in Figure 1C, the vibrational peak corresponding to -OH was observed at 3229 cm^−1^. Peaks at 2960 cm^−1^ and 2852 cm^−1^ corresponded to the stretching vibrations of C-H_2_, while peaks at 1630 cm^−1^, 1461 cm^−1^, and 1190 cm^−1^ corresponded to the deformation vibrations of the benzene ring C=C, the asymmetric vibrations of CH_3_, and the stretching vibrations of C-N, respectively. These peaks align with the functional groups of PDA, as reported in the literature [26], indicating the formation of the surface PDA coating. Peaks at 963 cm^−1^ and in the range of 1000–1200 cm^−1^ belong to the stretching modes of P-O, while peaks at 562 cm^−1^ and 603 cm^−1^ correspond to the bending vibrations of O-P-O. The peak at 630 cm^−1^ arises from the vibration mode of OH-, characteristic of hydroxyapatite, consistent with the functional groups of mHA reported in the literature [18], indicating the formation of the mHA coating. Taken together, these results demonstrate the successful preparation of PDA-mHA coating.

**Figure 2:**
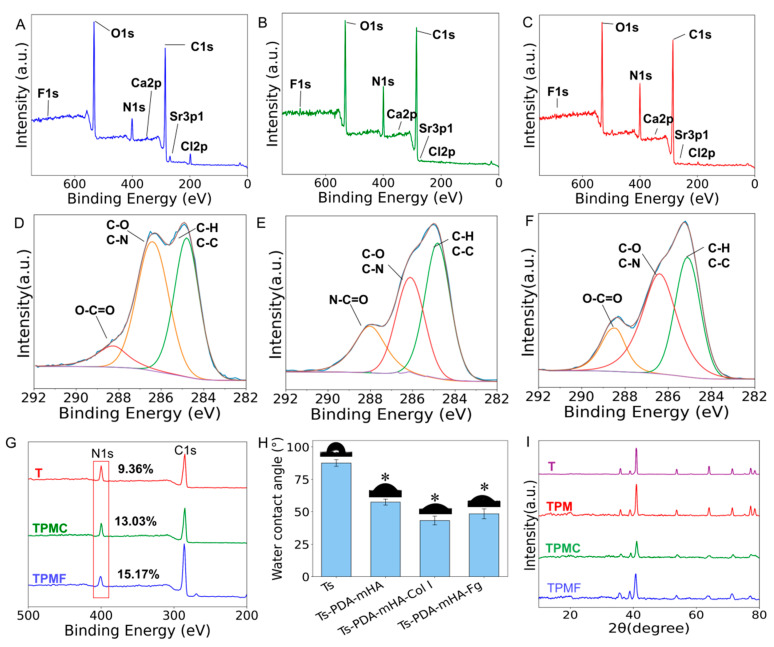
(**A**) Full spectrum of TPM. (**B**) Full spectrum of TPMC; (**C**) Full spectrum of TPMF. (**D**) C 1s spectrum of TPM. (**E**) C 1s spectrum of TPMC. (**F**) C 1s spectrum of TPMF. (**G**) Typical XPS spectra of TPM, TPMC, and TPMF groups. (**H**) Static water contact angles and corresponding images of the four groups, * *p* < 0.05 compared to Ts. (**I**) XRD patterns of the four sample groups.

Utilizing X-ray photoelectron spectroscopy (XPS) analysis (Figure 2A–F), characteristic peaks of type I collagen, such as N-C=O bonds, were observed in the C1s spectrum of TPMC. Additionally, the content of C=O double bonds in TPMF notably increased compared to the TMP group. Figure 2G illustrates that, relative to the surface of TPM, the nitrogen content on the surfaces of TPMC and TPMF increased after the deposition of Col I and Fg. The nitrogen content in the TPMC group rose from 9.36% to 13.03%, while in the TPMF group, it increased from 9.36% to 15.17%, indicating the successful introduction of Col I and Fg on the surface. Furthermore, no Ti 2p peaks originating from the titanium alloy substrate were observed in any of the three groups [24], suggesting uniform coating coverage over the substrate.

#### 3.2.3. Surface Hydrophilicity Testing

The hydrophilicity changes in different samples were evaluated using static water contact angle measurements. Figure 2H illustrates the static water contact angles and corresponding test photographs of four types of samples: Ts, Ts-PDA-mHA, Ts-PDA-mHA-Col I, and Ts-PDA-mHA-Fg. Analysis of the bar graph reveals that the water contact angles of the samples subjected to different treatments were significantly lower than that of the control group Ts (contact angle of Ts: 87.59° ± 2.58°, Ts-PDA-mHA: 57.4° ± 2.29°, Ts-PDA-mHA-Col I: 43.2° ± 3.39°, Ts-PDA-mHA-Fg: 48.39° ± 3.85°). This indicates that the addition of PDA-mHA, PDA-mHA-Col I, and PDA-mHA-Fg coatings caused the originally relatively hydrophobic titanium alloy surface to exhibit hydrophilicity, clearly reflecting the changes in surface properties.

#### 3.2.4. X-Ray Diffraction Analysis

To assess whether the addition of composite coatings affected the substrate’s phase, X-ray diffraction (XRD) analysis was conducted on the samples. As shown in Figure 2I, after modification with the three composite coatings, Ti-6Al-4V still exhibited peaks characteristic of Ti-6Al-4V, with the XRD patterns of each treatment group remaining largely consistent. This suggests that the addition of coatings did not have a significant impact on the substrate’s phase.

### 3.3. Coating Stability

**Figure 3:**
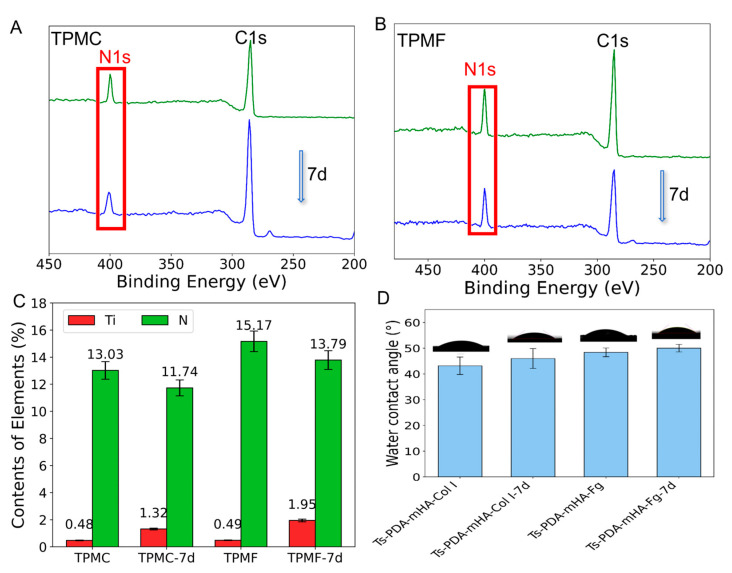
XPS spectra of (**A**) TPMC and (**B**) TPMF surfaces before and after immersion in PBS buffer for 7 days. (**C**) The bar graph shows the atomic ratios of nitrogen and titanium as measured by XPS. (**D**) Static water contact angles and corresponding optical images of the surfaces of Ts-PDA-mHA-Col I, Ts-PDA-mHA-Col I-7d, Ts-PDA-mHA-Fg, and Ts-PDA-mHA-Fg-7d samples.

To investigate the stability of PDA-mHA-Col I and PDA-mHA-Fg coatings, XPS analysis was performed on the TPMC and TPMF samples after 7 days of immersion. According to the data in Figure 3B, it can be observed that after 7 days of immersion, the nitrogen peak of TPMC-7d and TPMF-7d slightly decreased. Figure 3C provides specific data, showing a slight decrease in nitrogen content on the surface of TMPC from 13.03% to 11.74% and a slight increase in titanium content from 0.48% to 1.32%. The TPMF group exhibited a similar trend, with nitrogen content decreasing from 15.17% to 13.79% and titanium content increasing from 0.49% to 1.95%, indicating good stability of the PDA-mHA-Col I and PDA-mHA-Fg coatings. Subsequently, the difference in static water contact angles of Ts-PDA-mHA-Col I and Ts-PDA-mHA-Fg after 7 days of immersion was measured. As shown in Figure 3D, compared to the untreated samples, the static water contact angles of Ts-PDA-mHA-Col I-7d and Ts-PDA-mHA-Fg-7d increased slightly after 7 days of incubation in PBS, with angles of 46.02° ± 2.00° and 50.03° ± 3.60°, respectively, showing little difference from the untreated groups and confirming the relative stability of the coatings.

### 3.4. In Vitro Cells Experiments

#### 3.4.1. Cytotoxicity Assessment

**Figure 4:**
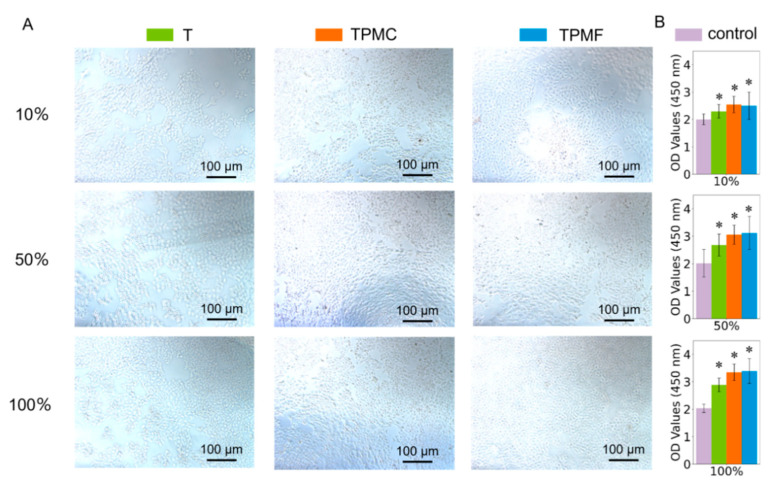
(**A**) Micrographs of HaCaT cells after 24 h of co-culture with the extract. (**B**) The CCK-8 assay results of HaCaT cells in different concentrations of extract from each sample (* *p* < 0.05 compared to the control group).

In vitro experiments were conducted according to the guidelines recommended in GB/T16886.5-2017 [27] for cytotoxicity assessment of biomaterials. The samples were co-cultured with human keratinocyte (HaCaT) cells for 24 h, and cell morphology was observed using microscopy. Cell viability was quantitatively assessed using a CCK-8 assay. The biocompatibility of different samples was compared. Figure 4A presents micrographs of HaCaT cells taken after 24 h of co-culture. The images indicate healthy cell growth in all treatment groups, particularly in the 50% and 100% extract concentrations of TPMC and TPMF samples, where cell density significantly increased compared to the control group (Group T). Figure 4B illustrates that, with the extract concentration controlled, cell viability significantly increased in the T, TPMC, and TPMF groups compared to the blank control group. Each sample exhibited a stable increase in cell viability with increasing extract concentration. The cell viability of the TPMC and TPMF groups surpassed that of the T and TPM groups, with the TPMF group demonstrating the highest cell viability, confirming the excellent biocompatibility of the scaffolds and coatings.

#### 3.4.2. Cell Adhesion and Spreading

**Figure 5:**
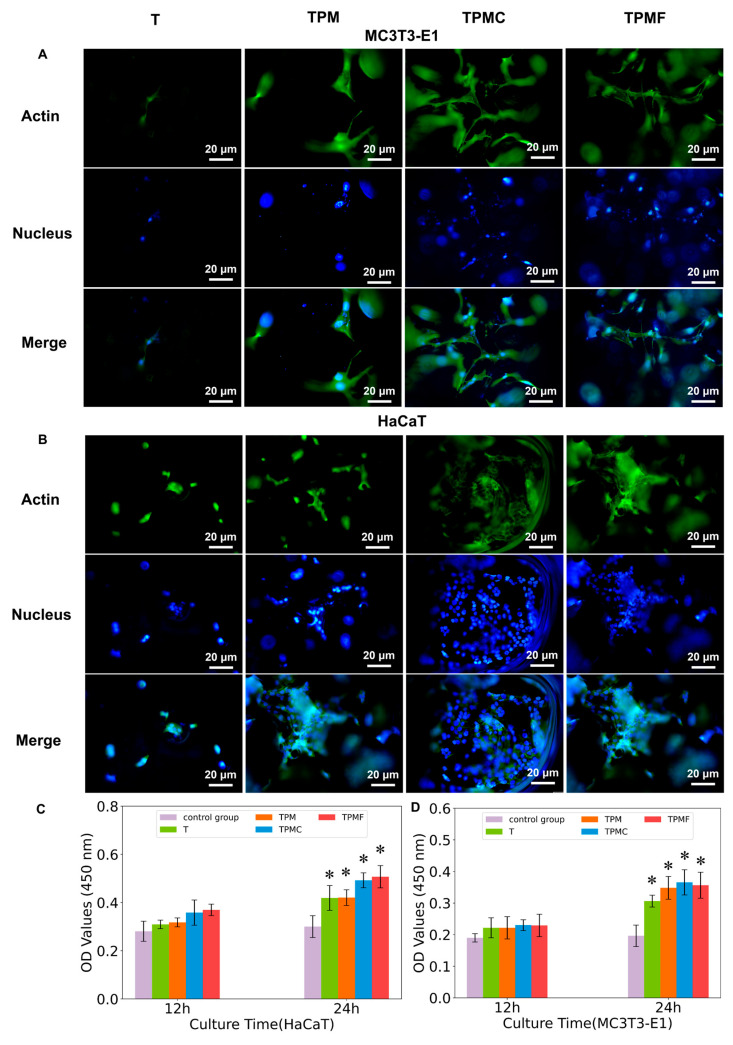
(**A**) The fluorescence microscopy images of preosteoblasts (MC3T3-E1). (**B**) Human keratinocyte cells (HaCaT) adhering and spreading on the surfaces of samples T, TPM, TPMC, and TPMF after 24 h of co-culture. The cell nuclei stained with DAPI appear blue, while the actin stained with FITC-phalloidin appears green. After 12 and 24 h of co-culture. (**C**) the CCK-8 assay results of human keratinocyte cells (HaCaT). (**D**) preosteoblasts (MC3T3-E1) adhering and spreading on the surfaces of the four groups are presented, with * *p* < 0.05 compared to T.

We investigated the adhesion and spreading of two cell types closely related to scaffold implantation, namely, keratinocytes (HaCaT) and preosteoblasts (MC3T3-E1). DAPI was used to label cell nuclei in blue, while FITC-phalloidin labeled actin in green. From Figure 5A, it is evident that the TPM, TPMC, and TPMF groups all exhibited enhanced cell adhesion, with the best adhesion observed in the TPMC and TPMF groups. Similarly, MC3T3-E1 cells showed improved spreading in the TPMC and TPMF groups, indicating a larger cell spreading area. CCK-8 results for adherent cell quantity indicated that the adhesion of HaCaT cells in the TPMC and TPMF groups was significantly higher than in other groups, with the TPMF group showing the best performance. Conversely, MC3T3-E1 cells exhibited the highest adhesion quantity in the TPMC group, suggesting that type I collagen had a superior early adhesion effect on osteoblasts compared to fibrinogen. Although the TPMF group demonstrated better cell spreading, there was no statistically significant difference in the adhesion quantity of MC3T3-E1 cells between the TPMF and TPM groups (*p* > 0.05), indicating no significant difference in their promotion of early osteoblast adhesion.

#### 3.4.3. Cell Proliferation

**Figure 6:**
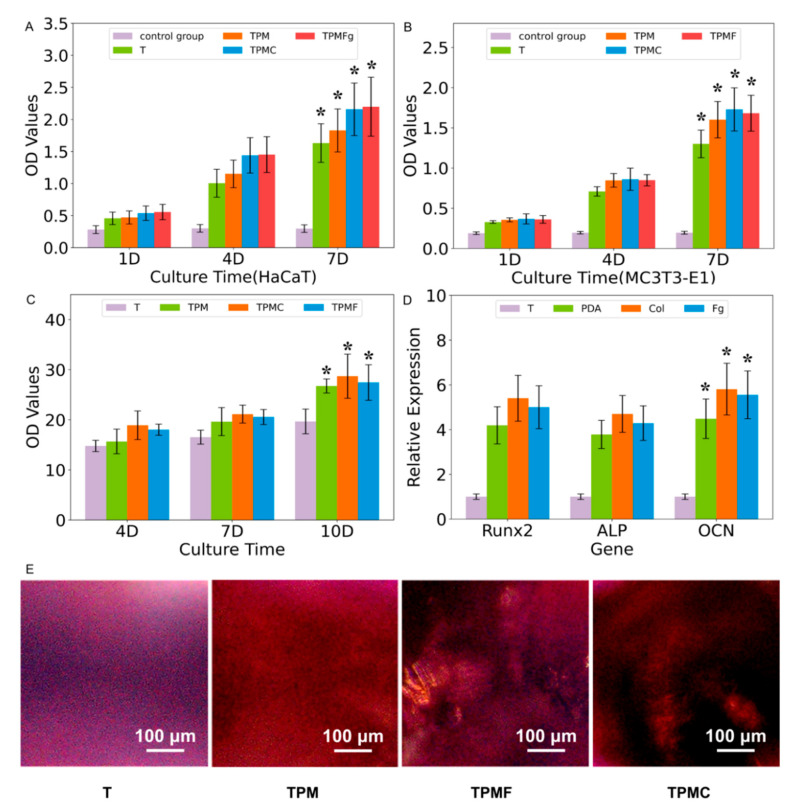
After co-culturing for 12h and 24h, the adhesion and spreading of (**A**) human keratinocytes (HaCaT) and (**B**) preosteoblasts (MC3T3-E1) on the surfaces of samples T, TPM, TPMC, and TPMF were assessed using CCK-8 assay, * *p* < 0.05 compared with T. (**C**) ALP activity of MC3T3-E1 cells co-cultured with each group for 4, 7, and 10 days,* *p* < 0.05 compared with T. (**D**) Relative expression levels of osteogenic genes Runx2, ALP, and OCN in MC3T3-E1 cells co-cultured with the samples for 14 days, * *p* < 0.05 compared with T. (**E**) Alizarin Red staining of the samples after 14 days of co-culture.

To assess the impact of various coatings on cell proliferation, the CCK-8 assay was employed to quantitatively evaluate the cell viability of human keratinocytes (HaCaT) and preosteoblasts (MC3T3-E1) co-cultured with T, TPM, TPMC, and TPMF samples on days 1, 4, and 7. The data presented in Figure 6A,B indicate that cell proliferation accelerated with increasing culture time, particularly with consistently higher proliferation rates observed in the TPM, TPMC, and TPMF treatment groups. Regarding the promotion of HaCaT cell proliferation, the TPM, TPMC, and TPMF treatment groups exhibited significant superiority over the T group (*p* < 0.05), with TPMC and TPMF outperforming TPM, and the most pronounced promotion observed in the TPMF group. In terms of MC3T3-E1 cell proliferation capacity, there were significant differences observed between the TPM, TPMC, TPMF, and T groups (*p* < 0.01), with significant differences also observed among the three treatment groups. These results demonstrate the significant promoting effects of TPM, TPMC, and TPMF on MC3T3-E1 cell proliferation, with the TPMC group showing the most favorable outcome.

#### 3.4.4. Alkaline Phosphatase Activity

To evaluate the osteogenic properties of various coatings, alkaline phosphatase (ALP) activity was initially assessed. ALP serves as a crucial indicator of early osteogenic differentiation in MC3T3-E1 cells [28]. By employing an alkaline phosphatase (ALP) activity assay kit, this study assessed the changes in ALP activity in preosteoblasts (MC3T3-E1) co-cultured with T, TPM, TPMC, and TPMF samples for 4, 7, and 10 days. As depicted in Figure 6C, the ALP activity of MC3T3-E1 cells exhibited an increasing trend with prolonged culture time. Compared to the T group as the control sample, the experimental groups (TPM, TPMC, TPMF) demonstrated significantly enhanced ALP activity, particularly in cells co-cultured with TPMC samples, which exhibited the strongest ALP activity, indicating the most robust promotion of early cell differentiation by the TPMC group. However, the difference between TPM and TPMF samples was not significant (*p* = 0.0859), suggesting that TPM, TPMC, and TPMF samples all promoted early osteogenic differentiation of MC3T3-E1 cells, with TPMC samples showing the most pronounced promotion effect, while the effects of TPMF and TPM were similar.

#### 3.4.5. Osteogenesis-Related Gene Expressions

Subsequently, after co-culturing the various sample groups with MC3T3-E1 cells for 14 days, the relative expression levels of the osteogenic-related genes Runx2, ALP, and OCN were assessed using RT-PCR. The results, as depicted in Figure 6D, revealed that the TPMC group exhibited the highest expression levels of all three genes, followed by TPMF, with TPM showing the lowest, yet all were significantly higher than the T group. This indicates that TPMC, TPMF, and TPM can significantly promote osteogenic cells, with TPMC demonstrating the most potent promotion effect.

#### 3.4.6. Alizarin Red S Staining

Alizarin Red S, a type of anthraquinone compound, forms an orange-red complex through chelation with calcium ions, suitable for assessing the presence of calcium nodules in the extracellular matrix. Alizarin Red S staining was employed to evaluate the impact of each group on the late-stage osteogenesis of MC3T3-E1 cells. In Figure 6E, the effects before and after Alizarin Red S staining of T, TPM, TPMC, and TPMF samples are compared. Notably, the surface of the MC3T3-E1 cells on TPMC samples exhibited the most significant depth of staining. This observation aligns with the earlier detection results.

## 4. Discussion

Three-dimensional printing technology enables the creation of porous scaffolds [29], and porous scaffolds change the mechanical properties of the scaffold, making it more similar to natural bone tissue, which makes it an ideal direction to replace autogenous bone. Additionally, ideal oral scaffolds should balance both osteogenic and soft tissue integration capabilities. Hydroxyapatite coatings are widely used in techniques promoting surface bone regeneration of scaffold materials and are a common choice. Early studies by our research group revealed that hydroxyapatite modified with multi-element doping (mHA) significantly enhanced the differentiation and bone formation of preosteoblasts (MC3T3-E1). Compared to traditional hydroxyapatite, this modified mHA not only exhibited a more stable crystal structure but also demonstrated superior bone conduction properties [18].

In order to enhance the osteogenic performance of 3D printed porous titanium alloy scaffolds, this study employed the self-polymerization properties of dopamine to form a PDA-mHA composite coating on the scaffold surface. As illustrated in Figure 1A, the titanium alloy scaffold was immersed in a suspension of dopamine and mHA. During the self-polymerization process, dopamine encapsulated mHA particles through its surface active groups, anchoring them onto the surface of the 3D printed porous titanium alloy, thereby forming the PDA-mHA composite coating [30].

Currently, a significant amount of research aims to enhance the osteogenic performance of scaffolds, yet there is limited focus on their soft tissue integration capability. Successful percutaneous scaffolding not only requires optimal bone integration but also necessitates the formation of a robust biological seal at the skin–scaffold interface, which is crucial for restoring skin barrier function and preventing bacterial colonization [24]. This underscores the necessity to improve the soft tissue integration performance of scaffolds. Therefore, to concurrently enhance both bone conduction performance and soft tissue integration capability, this study incorporated Type I collagen, which promotes fibroblast adhesion, induces greater synthesis of adhesion proteins, facilitates improved cell diffusion, and accelerates wound healing by enhancing wound integration and cell proliferation, onto the surface of TPM to form a PDA-mHA-Col I (Fg) composite coating. The formation of the coating is crucial for its stability, which is essential for the integration between the scaffold and soft tissues. Following scaffold implantation, enzymatic activity in bodily fluids, competition from other serum proteins, and physical wear within the body often lead to the depletion or removal of the coating [31,32]. Tissue regeneration and integration take time; therefore, the stability of the coating was assessed after 7 days of PBS immersion and rinsing treatment, which was confirmed through experiments measuring static water contact angle and XPS analysis, validating the formation of a stable composite coating. However, the concurrent effects of fibrinogen and collagen were not investigated in the present study. Whether these biomolecules can be co-immobilized to form a stable coating and simultaneously exert their intended biological functions represents a pertinent avenue for future in-depth investigation.

Utilizing techniques such as FE-SEM, static water contact angle testing, XRD, FTIR, and XPS, the characteristics of different samples were comparatively analyzed, encompassing the morphology of both scaffolds and coatings, the morphology of various coating components, hydrophilicity, as well as the composition of surface chemical elements and functional groups. The research findings indicate that the PDA-mHA-Col I (Fg) coating was not only successfully prepared but also demonstrated excellent stability, significantly enhancing the hydrophilicity of the scaffold surface. It is well established that the surface properties of scaffolds play a crucial role in promoting the adhesion, proliferation, and differentiation of osteoblasts, epidermal cells, and fibroblasts, directly impacting the efficiency of bone integration and soft tissue integration. The improvement in scaffold surface hydrophilicity facilitates the adhesion and proliferation of cells by promoting the attachment of relevant proteins and growth factors. Moreover, the mechanical properties of the scaffold, assessed through compressive testing, revealed that the compressive strength of the porous titanium alloy scaffolds fabricated in this experiment falls within the range of cortical bone compressive strength, and their elastic modulus is comparable to that of cortical bone, rendering them a feasible alternative to natural bone.

The manifestation of bone integration capability at the cellular level includes early cell adhesion, proliferation, and osteogenic differentiation; early cell adhesion on the scaffold fundamentally influences the subsequent remodeling of the extracellular matrix (ECM) and soft tissue regeneration, with human keratinocytes playing a significant role in the integration between the scaffold and soft tissues [32]. Therefore, this section utilized mouse preosteoblasts, namely MC3T3-E1 cells, to represent osteogenic capability and also employed human keratinocytes, HaCaT cells, which play a crucial role in soft tissue integration, to demonstrate soft tissue integration capability. A combined approach of quantitative detection and qualitative observation was utilized to comprehensively assess the impact of porous 3D printed titanium alloy scaffolds with PDA-mHA-Col I (Fg) composite coatings on bone integration and soft tissue integration capability.

Initially, the cytotoxicity testing revealed excellent biocompatibility across all sample groups, a result further confirmed by multi-day cell proliferation experiments. Among these, the TPMC and TPMF groups exhibited significantly higher cell proliferation rates than the T group, and in terms of HaCaT cell proliferation, they surpassed the TPM group, affirming the coating’s ability to promote soft tissue cell proliferation, aligning with the cell adhesion outcomes. Cell adhesion marks the first biological behavior of cells implanted on scaffolds and serves as the foundation for cell proliferation, migration, and differentiation [21]. Cell adhesion results indicated a notable increase in the number of HaCaT and MC3T3-E1 cells adhering to the surfaces of the experimental groups TPM, TPMC, and TPMF compared to T, with enhanced cell spreading. These findings corresponded with the results of the static water contact angle tests for each group, underscoring the crucial role of surface coatings in place. For HaCaT cells, the adhesion in the TPMC and TPMF groups notably exceeded the first two groups, in line with the effects of Col I and Fg additions. Regarding MC3T3-E1 cells, the adhesion in the TPM group was slightly lower than in the TPMC and TPMF groups, with marginal differences. This not only affirmed the positive impact of PDA-mHA coatings on bone cell adhesion and proliferation [33] but also demonstrated that the inclusion of Col I and Fg did not adversely affect the osteogenic capability of the original PDA-mHA coating. On the contrary, it exhibited a certain promotional effect, consistent with reported literature results [21,24].

In terms of bone integration, cellular osteogenic differentiation plays a crucial role. Alkaline phosphatase (ALP), as one of the vital markers for early cellular osteogenic differentiation, reflects the progression from early to late osteogenic differentiation through the formation of calcium nodules. The results of this study showed that compared to sample T, cells co-cultured with samples TPM, TPMC, and TPMF exhibited higher ALP activity, with the highest activity observed in cells co-cultured with sample TPMC, consistent with the observations from Alizarin Red staining. However, there was no statistically significant difference in ALP activity between the TPM and TPMF groups, indicating that Fg within the composite coating may not strongly promote early bone integration, while Col I may have a certain promoting effect on bone integration. In this study, real-time quantitative polymerase chain reaction (RT-qPCR) was partially utilized to evaluate the expression levels of osteogenic key genes Runx2, ALP, and OCN within MC3T3-E1 cells after 14 days of co-culture with different experimental group samples. This work aimed to gain a deep understanding of the influence of materials on cellular osteogenic differentiation capability by precisely determining the relative expression levels of these genes. Runx2, essential for osteoblast differentiation and bone formation, regulates the expression of other osteogenic-related genes such as Osteocalcin (OCN) [34]. ALP, an extracellular enzyme, is highly expressed in active osteoblasts and plays a role in bone mineralization by controlling the concentration of mineralization inhibitors and phosphate ions [35], serving as an early marker for cellular osteogenic differentiation. OCN is the second most abundant protein in bone tissue (second only to collagen) and promotes mineral deposition, mainly found in fully mineralized matrix, serving as a late marker for cellular osteogenic differentiation [36]. From the experimental results, it can be observed that compared to the T group, the expression of Runx2, OCN, and ALP in the TPM, TPMC, and TPMF groups was significantly upregulated, with TPMC group showing higher relative expression levels than other groups, indicating that mHA, Col I, and Fg can all promote osteogenic differentiation of MC3T3-E1 cells, with the combination of mHA and Col I demonstrating relatively better effects, consistent with the expected results, while the effect of PDA-mHA coating in the TPM group is similar to the results reported by Shen et al. [37]. In the in vitro cellular assays conducted herein, the removal of non-adherent or loosely adherent cells was primarily achieved by multiple PBS (phosphate-buffered saline) rinses of the well plates. It is acknowledged that the direct replacement of the well plates following specific incubation periods might yield more precise quantitative data. Furthermore, for the assessment of cytocompatibility and cell proliferation, the incorporation of live/dead staining assays is recommended to provide a more comprehensive evaluation of cell viability. Concurrently, acquiring photomicrographs of cell populations at various time points throughout the proliferation assays would offer a more robust visual and qualitative assessment of cell growth dynamics.

This study demonstrates the significant enhancement of cell adhesion and proliferation of HaCaT cells achieved by utilizing dopamine’s self-polymerizing properties to form a PDA-mHA-Col I (Fg) composite coating on 3D printed porous titanium alloy scaffolds. Furthermore, this coating further augmented the promotion of cell adhesion, proliferation, and osteogenic differentiation of MC3T3-E1 cells, aligning with the initial research expectations. The incorporation of mHA increased the scaffold’s bonding strength with bone tissue, while the biological activity of Col I and Fg enhanced the scaffold’s integration capabilities for soft tissue. The discovery and application of polydopamine provided great convenience in the preparation of this composite coating, as it not only simplifies the preparation process and enhances operability but also exhibits excellent compatibility with porous structures. Given that implanted materials and their associated coatings encounter a complex physiological environment in vivo, long-term implantation studies are imperative. Such studies are essential to rigorously evaluate their in vivo stability, degradation kinetics, and the biocompatibility of any degradation byproducts. Moreover, future research endeavors should encompass in vivo assessments of osseointegration efficacy and the capacity to promote effective soft tissue sealing at the implant interface.

## 5. Conclusions

In this study, porous Ti-6Al-4V scaffolds were prepared using selective laser melting technology. They exhibited a porosity ranging from 54.0% to 58.0% and an elastic modulus between 19.4 and 23.3 GPa, which closely resembled the elastic modulus of cortical bone. Then the scaffolds were immersed into a mixed solution containing polydopamine and multi-doped hydroxyapatite (mHA) to obtain a PDA-mHA coating. Subsequently, the TPM samples were immersed in solutions of type I collagen and fibrinogen, resulting in the successful fabrication of corresponding scaffolds with PDA-mHA-Col I and PDA-mHA-Fg composite coatings. The cytotoxicity experiments demonstrated good biocompatibility of the scaffolds and the composite coatings. The adhesion, spreading, and proliferation of HaCaT and MC3T3-E1 cells were promoted by the TPM, TPMC, and TPMF scaffolds. The mHA coating primarily enhanced the activity of bone cells, while the Col I and Fg coatings mainly promoted the activity of soft tissue cells. The research provides useful information for surface modification of Ti-6Al-4V-based biomedical implants and a promising method for improving soft tissue integration ability for implants.

## Figures and Tables

**Table 1:** Preparation solution of PDA coating and PDA-mHA coating.

Groups	DA-Tris (mg/mL)	mHA-Tris(mg/mL)
T-PDA (TP)	2	-
T-PDA-mHA (TPM)	2	4

**Table 2:** Primer sequence for RT-PCR.

Gene	Sequence
*GADPH*	Upstream: GAGTCGGTGTGAACGGATTTG
	Downstream: TGTAGACCAGTTAGTTGAGGTCA
*ALP*	Upstream: CACGGCGTCCATGAGCAGAAC
	Downstream: CAGGCACAGTGGTCAAGGTTGG
*RunX2*	Upstream: ATTGCAGGCTTCGTGGTTGAGG
	Downstream: TGGCTGTATGGTGAGGCTGGTAG
*OCN*	Upstream: GCTCGGCTTTGGCTGCTCTC
	Downstream: AGCTGCTGTGACATCCATACTTGC

## Data Availability

The data that support the findings of this study are available from the corresponding author upon reasonable request.
